# The prevalence of the *AR-V7* variant and its association with clinicopathologic characteristics in non-prostatic cancers—a systematic review

**DOI:** 10.1093/carcin/bgag052

**Published:** 2026-08-04

**Authors:** Zaineh Alnoubani, Youssuf Khanafer, Adnan Fojnica, Emir Begagic, Inga Rose, Zoran Gatalica, Semir Vranic

**Affiliations:** Department of Basic Medical Sciences, College of Medicine, QU Health, Qatar University, PO Box 2713, 2713 Doha, Qatar; Department of Basic Medical Sciences, College of Medicine, QU Health, Qatar University, PO Box 2713, 2713 Doha, Qatar; Institute of Virology, TUM School of Medicine, Technical University of Munich, Trogerstraße 30, 81675 Munich, Germany; Department of Neurosurgery, Cantonal Hospital Zenica, Crkvice 67, 72000 Zenica, Bosnia and Herzegovina; Reference Medicine, 4050 E Cotton Center Blvd Bldg 3 Suite 38, 85040 Phoenix, AZ, United States; Reference Medicine, 4050 E Cotton Center Blvd Bldg 3 Suite 38, 85040 Phoenix, AZ, United States; Department of Basic Medical Sciences, College of Medicine, QU Health, Qatar University, PO Box 2713, 2713 Doha, Qatar

**Keywords:** *AR-V7*, non-prostatic cancers, androgen receptor, therapeutic resistance, systematic review

## Abstract

Androgen receptor splice variant 7 (*AR-V7*) is associated with resistance to androgen receptor-targeting therapies in prostate cancer, but its prevalence and clinical relevance in non-prostatic cancers remain incompletely characterized. A systematic review was conducted in accordance with PRISMA guidelines. Thirty-four studies, including 4855 clinical cases and 60 cell lines, were included. Overall, *AR-V7* positivity was reported in 948/4855 clinical cases (19.5%); however, this represents a descriptive estimate across heterogeneous tumor types, study designs, and detection methods. Breast cancer accounted for 4057 of 4855 clinical cases (83.6%) and 815 of 948 *AR-V7*-positive cases (86.0%), with a within-cancer prevalence of 20.1%. However, after excluding the TCGA cohort in which *AR-V7* was inferred through splice-junction analysis, the prevalence of *AR-V7*-positive breast cancer decreased to 9%. Higher tumor-specific proportions were observed in salivary duct carcinoma (55.2%), hepatocellular carcinoma (57.1%), and non-muscle-invasive bladder cancer (82.6%), but these estimates were based on smaller cohorts and should be interpreted cautiously. *AR-V7* was present in several treatment-naive non-prostatic cancers, suggesting that it may represent a preexisting molecular feature in selected contexts. Current evidence suggests that *AR-V7* warrants further investigation as a candidate biomarker. Standardized detection methods and prospective studies are needed before its clinical utility can be established.

## Teaser

*AR-V7* is detectable in multiple non-prostatic cancers, with variable prevalence across tumor types. Unlike prostate cancer, it often appears in treatment-naive cancers, indicating a possible intrinsic role in anti-AR resistance.

## Graphical Abstract

**Graphical Abstract:**
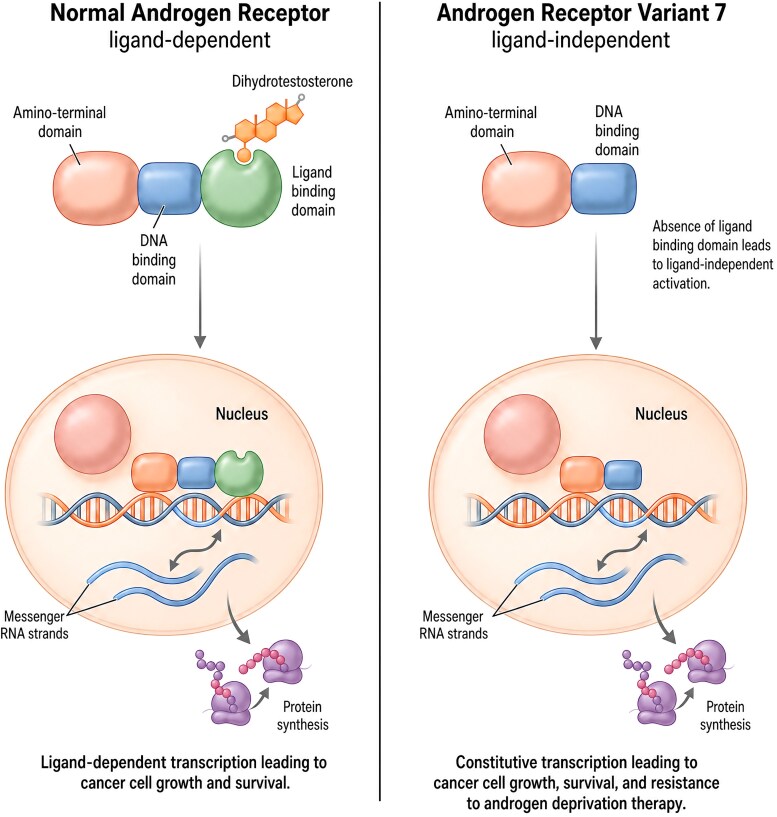

## 1. Introduction

The androgen receptor (AR) is a nuclear receptor that binds androgens such as testosterone and dihydrotestosterone. Upon activation through androgen binding, the AR translocates to the nucleus and regulates gene transcription, thereby modulating various physiological processes. These physiological processes differ based on sex and age. In men, AR signaling is essential for prostate development and function, as well as spermatogenesis and testosterone production, contributing to overall fertility [1]. In women, AR is expressed widely in the ovary, uterus, and breast, playing a central role in follicular development, ovulation, and mammary gland growth [2].

AR signaling is a major driver of prostate cancer (PCa) [3]. Although androgen deprivation therapy (ADT) is initially effective, many patients eventually develop castration-resistant prostate cancer (CRPC), typically within 5 years of follow-up, prompting investigation into mechanisms of therapeutic resistance [4].

Different variants of AR can be expressed, either innately or secondary to ADT resistance, as seen in PCa. These variants fall into several categories, mainly splice variants, point mutations, and copy number variations [5]. The most clinically significant AR splice variants lack the ligand-binding domain (LBD) and are more profoundly expressed in CRPC. Variants lacking the LBD, such as *AR-V1, AR-V7,* and *AR-V567es*, are constitutively active and correlate with disrupted cell-cycle regulation, leading to resistance development and poor clinical outcomes [6, 7]. Among these variants, *AR-V7* is one of the most prevalent and clinically significant AR variants identified in CRPC [7–9]. *AR-V7* arises through alternative splicing that removes the LBD, enabling ligand-independent transcriptional activity. *AR-V7*’s loss of the LBD allows it to evade inhibition by agents targeting this region, including enzalutamide and abiraterone [7, 9, 10]. As such, *AR-V7* has become an established predictive biomarker in metastatic CRPC and has been associated with resistance to several anti-androgen therapies [11]. Guidelines now recognize *AR-V7* testing in circulating tumor cells (CTCs) as a clinically validated predictive tool to guide therapy selection in metastatic CRPC [11].

*AR-V7* is gaining recognition in non-prostatic cancers, particularly in breast cancers (BrCa) and salivary duct carcinomas (SDC). Clinical studies have shown that *AR-V7* expression in BrCa is associated with more aggressive disease and higher tumor grades, further supporting its potential as a prognostic biomarker and therapeutic target [12]. Moreover, the presence of *AR-V7* in 48% of SDC cases suggests a potential role for refining patient selection for hormonal therapies [13]. However, the functional role of *AR-V7* in BrCa, SDC, and other non-prostatic cancers remains incomplete and requires further investigation.

This study aimed to summarize the current evidence on the prevalence and clinical significance of *AR-V7* in non-prostatic cancers. Specifically, the study summarized reported *AR-V7* prevalence across different tumor types, reviewed the available clinical and preclinical evidence regarding its potential associations with disease characteristics and treatment response, and described the methodologies used to detect *AR-V7* across studies. It also highlighted methodological heterogeneity among detection approaches and discussed its implications for reported prevalence estimates and clinical interpretation [e.g. immunohistochemistry (IHC), polymerase chain reaction (PCR)-based assays, and RNA sequencing-based approaches].

## 2. Methods

### 2.1. Study design

This systematic review followed the Preferred Reporting Items for Systematic Reviews and Meta-Analyses (PRISMA) guidelines (Supplementary File S1). The protocol for this review is registered on the International Prospective Register of Systematic Reviews (PROSPERO) (CRD420251083307).

### 2.2. Data sources and search methods

For this study, a comprehensive literature review was conducted using established electronic databases. We searched PubMed/MEDLINE/PMC, SCOPUS, Cochrane Library, CINAHL Ultimate, and the Web of Science Core Collection, covering all available records until the end of October 2025. Additionally, proceedings from major cancer/oncology/pathology scientific conferences, such as the American Society of Clinical Oncology (ASCO), the European Society for Medical Oncology (ESMO), the United States and Canadian Academy of Pathology (USCAP), and the American Association for Cancer Research (AACR), were also explored. Search terms related to both *AR-V7* and non-prostatic cancers were used to identify relevant research. The search terms used are provided in Supplementary File S2.

### 2.3. Eligibility criteria

Eligibility for this systematic review was defined using the Patient/Population/Intervention/Comparison/Outcome/Study Design (PICOS) framework. The Patient/Population included individuals with a confirmed diagnosis of any non-prostatic cancer, at any stage. The Intervention was the assessment of *AR-V7* expression. A comparator was not required for the primary objective of determining prevalence; however, for secondary analyses, comparisons between *AR-V7-*positive and *AR-V7-*negative tumors were considered. The primary outcome was the prevalence of *AR-V7* among non-prostatic cancers, while secondary outcomes included associations with tumor subtype, patient survival outcomes, and response to treatment. Eligible study designs included all primary research formats, case reports, case series, cohort studies (prospective and retrospective), and clinical trials. In addition, preclinical studies investigating the regulation, functional role, or therapeutic modulation of *AR-V7* in human cancer cell models were reviewed narratively to contextualize mechanistic findings in the literature. Animal *in vivo* studies were excluded. Conference abstracts and proceedings were also included if they provided sufficient data on cancer type, *AR-V7* detection method, and *AR-V7* prevalence.

### 2.4. Study selection

Following the database searches, all retrieved records were imported into EndNote software for deduplication. The remaining references were then uploaded to the Rayyan systematic review management platform (https://www.rayyan.ai/) for blinded title and abstract screening by two independent reviewers. After unblinding, screening decisions were compared, and discrepancies were resolved through consultation with a third reviewer (S.V.). Studies meeting the inclusion criteria were subsequently subjected to full-text screening, which was performed independently and in a blinded manner by two reviewers. Any conflicts were resolved by a third reviewer (S.V.).

### 2.5. Key definitions

- Androgen receptor splice variant 7 (*AR-V7*)

*AR-V7* is a truncated isoform of AR generated through alternative splicing of the AR pre-mRNA. *AR-V7* retains the N-terminal transactivation domain and DNA-binding domain but lacks the C-terminal LBD, rendering it constitutively active and independent of androgen stimulation [10].

Non-prostatic cancer

Non-prostatic cancer refers to any histologically confirmed malignant neoplasm originating outside the prostate gland, irrespective of AR expression status, hormone sensitivity, or tissue lineage.

### 2.6. Outcomes

The primary outcome of this systematic review was to determine the prevalence of *AR-V7* expression in non-prostatic cancers across different tumor types. Secondary outcomes included the prevalence of wild-type AR and the methods used for *AR-V7* detection. Additionally, we aimed to assess the clinical significance of *AR-V7* expression in relation to tumor progression, treatment resistance, and survival outcomes. In parallel, we prespecified a narrative synthesis of mechanistic outcomes, including the regulation of *AR-V7* (up- and down-regulators) and the functional impact of *AR-V7* on AR signaling and therapeutic resistance in preclinical models. We also reviewed findings from studies evaluating the impact of anti-androgen agents (e.g. enzalutamide and abiraterone) on *AR-V7*-positive non-prostatic cancers.

### 2.7. Data extraction

Data extraction was performed independently by two reviewers using a standardized Microsoft Excel template. Extracted variables included study characteristics (title, author, publication year, study design, study duration, and country), patient demographics (age, gender), cancer-related data (cancer type, histological subtype, and tumor site), and *AR-V7* findings (presence or absence of *AR-V7*, presence or absence of AR, co-expression of other AR variants, and the method used to detect *AR-V7*). Patients were included only if they had a non-prostatic cancer and had their *AR-V7* status investigated. Treatment modality and clinical outcomes (mortality, recurrence or progression, response status, and follow-up duration) were also collected. For studies involving *in vitro* models, mechanistic and regulatory findings related to *AR-V7* were narratively extracted. After extraction, the two authors compared entries to ensure accuracy and consistency. A third reviewer (Z.A.) independently reviewed the extracted dataset to identify potentially overlapping patient cohorts. This assessment was performed by comparing study characteristics, including author lists, institutions, recruitment periods, data sources (e.g. institutional cohorts, publicly available datasets, and commercial molecular profiling datasets), patient demographics, sample size, tumor subtype, and other cohort characteristics. The full texts were also reviewed to identify explicit statements indicating that a patient cohort had been previously reported. Where overlapping cohorts were identified, duplicate patient data were excluded, and only the dataset contributing unique patient information was retained for analysis.

### 2.8. Data synthesis

A narrative synthesis was conducted to summarize the prevalence of *AR-V7* expression across different non-prostatic cancers. Findings were summarized in structured tables to describe study characteristics, detection methods, *AR-V7* and AR positivity rates, and reported associations with treatment response, disease progression, and survival outcomes. Mechanistic evidence from *in vitro* studies was synthesized thematically to characterize the regulation of *AR-V7* expression, its functional role in AR signaling, and its potential implications for therapeutic resistance. Due to substantial heterogeneity in study designs, outcome definitions, and reporting formats, quantitative pooling of results was not undertaken. To evaluate the robustness of the descriptive prevalence estimates, sensitivity analyses were performed where appropriate.

### 2.9. Quality and risk of bias assessment

The methodological quality and risk of bias of the included studies were evaluated using appraisal instruments matched to their study designs. Observational studies reporting the prevalence of *AR-V7* in non-prostatic cancers were assessed with the Joanna Briggs Institute (JBI) Critical Appraisal Checklist for Studies Reporting Prevalence Data [14]. Case reports (and, where applicable, case series) were appraised using the corresponding JBI critical appraisal checklists [15]. Randomized controlled trials were assessed with the Cochrane Risk of Bias 2 (RoB 2) tool, applying the domain-based signaling questions and decision rules specified in the RoB 2 guidance [16].

Preclinical *in vitro*/cell-line studies were evaluated using an adapted reproducibility-focused checklist emphasizing: (i) cell line identity verification (e.g. STR-based authentication), (ii) routine mycoplasma testing, (iii) use of biological and technical replicates, (iv) adequacy of experimental controls, (v) analytical validity and transparent reporting of *AR-V7* detection assays, (vi) completeness of statistical reporting, and (vii) transparency indicators (e.g. data/material availability and conflict-of-interest declarations), aligned with established good practice and standards for cell-based research [17, 18]. For all appraisal tools, each item was rated Yes/No/Unclear/Not applicable based on information reported in the publication. Items insufficiently described were coded as Unclear. An overall study-level judgement (low/moderate/high risk of bias) was derived using a prespecified decision rule combining the number of affirmative items with the presence of critical flaws (e.g. an inadequately described *AR-V7* detection method or inconsistent denominators). Any disagreements were resolved by consensus.

Finally, to minimize potential bias, studies involving any current author and/or affiliated institutions were not assessed by the potentially conflicted authors. Data extraction, eligibility assessment, and quality/risk-of-bias evaluation for these studies were performed or verified by co-authors who were not involved in the original studies.

## 3. Results

### 3.1. Search results

**Figure 1:**
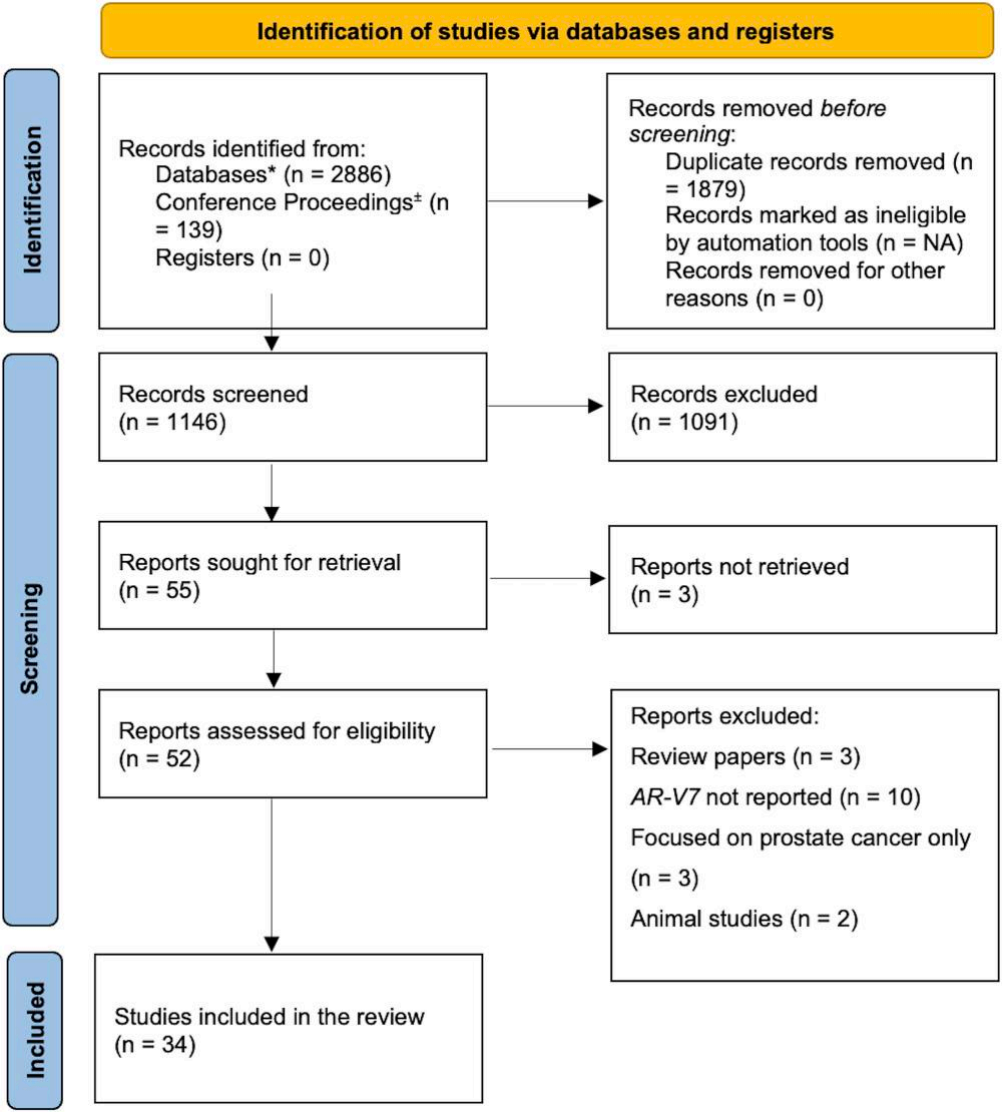
Preferred Reporting Items for Systematic Reviews and Meta-Analyses (PRISMA) flowchart for the study. *Databases used were PubMed/MEDLINE, SCOPUS, Web of Science Core Collection (WoS), CINAHL Ultimate, and Cochrane Library. ^±^Conference proceedings searched were those of the American Society of Clinical Oncology (ASCO), the European Society for Medical Oncology (ESMO), the American Association for Cancer Research (AACR), and the United States and Canadian Academy of Pathology (USCAP). NA: not applicable; AR-V7: androgen receptor splice variant 7.

The study selection process is shown in the PRISMA flow diagram (Fig. 1). A total of 2886 records were identified through database searches, with an additional 139 conference abstracts identified through conference proceedings. Following deduplication, 1146 unique records underwent title and abstract screening, of which 55 were deemed eligible for full-text assessment. Full texts could not be retrieved for three records, leaving 52 studies for full-text review. Of these, 3 were excluded because they were review articles; 10 did not report *AR-V7* prevalence; 3 focused exclusively on PCa; and 2 were animal studies. Ultimately, 34 studies [12, 13, 19–50] met the eligibility criteria and were included in the final analysis (Supplementary Table S1), 6 of which [21, 23, 39, 43, 49, 50] were conference abstracts without corresponding full-text publications.

### 3.2. Quality and risk-of-bias assessment of included studies

Across the 34 included studies, overall methodological quality was predominantly moderate-to-high risk of bias (3/34 low [31, 40, 42], 16/34 moderate [12, 13, 21–23, 25, 26, 28–30, 33, 36, 38, 45, 46, 49], 15/34 high [19, 20, 24, 27, 32, 34, 35, 37, 39, 41, 43, 44, 47, 48, 50]). Among clinical prevalence/observational studies (*n* = 17) [13, 21–23, 26, 29, 30, 32, 33, 36–40, 43, 45, 50], most were rated moderate risk (*n* = 11/17) [13, 21–23, 26, 29, 30, 33, 36, 38, 45], with 5/17 being high risk [32, 37, 39, 43, 50] and 1/17 being low risk [40]. Ratings were chiefly driven by limited reporting of sampling representativeness (*n* = 15/17 unclear) and response/coverage (*n* = 17/17 unclear), together with frequent concerns regarding sample size adequacy (*n* = 12/17 inadequate). All prevalence studies met the JBI criterion for the use of valid methods to identify the condition (*n* = 17/17); however, substantial heterogeneity in *AR-V7* detection methods persisted across studies. Preclinical studies (*n* = 14) [12, 19, 20, 24, 25, 27, 31, 35, 41, 42, 44, 46–48] were largely high risk (*n* = 9/14) [19, 20, 24, 27, 35, 41, 44, 47, 48], with 3/14 being moderate [12, 25, 46], and 2/14 being low risk [31, 42], primarily due to incomplete reporting of key reproducibility elements (e.g. cell line authentication, mycoplasma testing, controls, statistical reporting, and data availability). The only trial included in this review demonstrated a high risk of bias [34]. This was largely attributable to incomplete outcome data, which limited the internal validity of the trial and constrained the interpretability of its findings. Consequently, the interventional evidence base remains limited. The case report [28] and case series [49] were rated as moderate risk. Overall, the evidence supports *AR-V7* detectability across settings, but limits confidence in precise prevalence estimates and inferences regarding treatment effects. Detailed quality and risk-of-bias assessment results for all included studies are provided in Supplementary File S3.

### 3.3. Characteristics of included studies

Thirty-four studies [12, 13, 0] included 60 cell lines and 4855 unique clinical cases. Overall, the studies encompassed heterogeneous patient populations and tumor types, reflecting the growing interest in the role of *AR-V7* beyond PCa. The studies were published between 2014 and 2025. Eighteen studies were conducted exclusively in the United States of America [12, 13, 20, 22–26, 29, 30, 32, 34, 38–40, 42, 43, 49], while five additional studies involved the United States in collaboration with institutions from other countries [31, 37, 48, 50]. The remaining studies were conducted across various countries worldwide. Further details are provided in Table 1.

**Table 1:** Characteristics of included studies.

No.	Author, year	Country	*AR-V7* detection method	Total cases of non-prostatic cancer	Total non-prostatic cancer cell lines	No. of pts in each type of cancer	Total *AR-V7-*positive cases	Total *AR-V7-*positive cell lines
1	Hu, 2014 [19]	Australia	RT-qPCR	0	7	MFM223, MDA-MB-453, MDA-MB-231, ZR75.1, MCF7, T47D, HepG2	0	7
2	Mitani, 2014 [20]	USA	RT-qPCR, IHC, WB, FISH, DNA-seq	35	2	35 SDC, one RET981, one A253	13	NM
3	Hickey, 2015 [12]	USA	RNA-seq, IHC, WB, RT-qPCR, dual-label immunofluorescence	1111	8	1111 BrCa, MFM-223, MDA-MB-453, ZR-75-1, T47D, MDA-MB-231, MCF7, CAL-51, CAL-148	573	6
4	Asano, 2016 [21]^a^	Japan	IHC	177	0	177 BrCa	43	0
5	Dalin, 2016 [22]	USA	WES, RNA-seq, IHC, NGS, PCR	16	0	16 SDC	8	0
6	Singh, 2016 [23]^a^	USA	IHC, RT-qPCR	138	0	138 BrCa	25	0
7	Stossi, 2016 [24]	USA	IHC, WB, RT-qPCR, FISH, DNA-seq	0	3	One MCF-7, one LLC2 (MCF-7 Tamoxifen-resistant), and one T47D	0	3
8	Li, 2017 [25]	USA	RT-qPCR, IHC, WES	1	0	One primary SDC	1	0
9	Aceto, 2018 [26]	USA	RNA-seq, IHC	22	0	22 BrCa	9	0
10	Braga, 2018 [27]	Brazil	RT-qPCR	0	2	MCF7, MDA-MB-231	0	2
11	Cappelletti, 2018 [28]	Italy	RT-qPCR	1	0	One SDC	1	0
12	Kang, 2018 [29]	USA	RISH assay	8	0	Eight SDC	2	0
13	Gargano, 2019 [13]	USA	IHC, NGS	27	0	27 SDC	13	0
14	Yang, 2019 [30]	USA	RISH, IHC, RT-qPCR	23	0	23 SDC	14	0
15	Dauki, 2020 [31]	USA, Egypt	RNA-seq, RT-qPCR, WB, WGS, immunofluorescence	28	6	28 HCC, HepG2, PLC/PRF/5, SNU-423, HCCLM3, HepG2/C3A, SNU-475	16	3
16	Gatalica, 2020 [32]	USA	IHC, NGS	28	0	18 primary extra-mammary Paget's disease, 10 mammary Paget's disease	2	0
17	Kasimir-Bauer, 2020 [33]	Germany	RT-qPCR	41	0	41 BrCa	8	0
18	Lehmann, 2020 [34]	USA	RNA-seq, DNA-seq	8	0	Eight BrCa	5	0
19	Song, 2020 [35]	China	RT-qPCR, WB, IHC, RNA-seq	0	5	HCCLM3, PLC5, Huh7, HepG2, SMMC-7721	0	NM
20	van Boxtel, 2020 [36]	The Netherlands	RT-qPCR	44	0	44 SDC	41	0
21	Kruslin, 2021 [37]	USA, Croatia, Czech Republic, Qatar, Bosnia/Herzegovina	IHC, NGS	29	0	Seven metastatic testicular LCTs, 20 primary testicular LCTs, one metastatic ovarian LCT, one primary ovarian LCT	0	0
22	Schvartsman, 2021 [38]	USA	IHC	17	0	17 SDC	2	0
23	Wilson, 2021 [39]^a^	USA	RNA-seq	11	0	11 HGSOC	0	0
24	Ferguson, 2022 [40]	USA	MSK-Fusion targeted RNA-seq, MSK-IMPACT targeted DNA-seq, IHC if sequencing was positive	196	0	196 BrCa	19	0
25	Ali, 2023 [41]	Egypt	Immunocytochemistry, WB, ELISA	0	1	T47D	0	1
26	Asemota, 2023 [42]	USA	RT-qPCR, IHC, WB	52	4	52 BrCa, MDA-MB-453, MDA-MB-231, BT549, MFM223	6	0
27	Gatalica, 2024 [43]^a^	USA, Qatar	OncoExTra test, a tumor-normal, whole-exome, whole-transcriptome assay.	1944	0	1944 BrCa	20	0
28	Kuroiwa, 2024 [44]	Japan	RT-qPCR, WB, immunofluorescent staining	0	18	MDA-MB-157, MDA-MB-231, MDA-MB-468, HCC38, Hs578T, BT-549, MDA-MB-453, UACC-893, HCC202, SK-BR-3, HCC1954, HCC1419, MDA-MB-361, UACC-812, BT-474, ZR-75-1, T47D, MCF7	0	7
29	Sarıkaya, 2024 [45]	Turkey	RT-qPCR, WB	23	0	23 treatment-naive NMIBC	19	0
30	Srivastava, 2024 [46]	India	RT-qPCR, WB, IHC	76	3	76 BrCa, MDA-MB-231, MCF7, MDA-MB-453	52	3
31	Ali, 2025 [47]	Egypt	IHC, bioinformatics analysis of RNA status, ELISA	251	1	251 BrCa, MDA-MB-231	49	1
32	Bian, 2025 [48]	USA, China, Sweden	SplitFusion algorithm on RNA-seq and anchored multiplex PCR	515	0	515 lung adenocarcinomas	0	0
33	Wang, 2025 [49]^a^	USA	RNA-seq	2	0	Two gynecologic cancers	2	0
34	Gatalica, 2025 [50]^a^	USA, Bosnia/Herzegovina, Qatar	NGS, IHC	31	0	31 BrCa	5	0

*AR-V7*, Androgen receptor splice variant 7; BrCa, breast cancer; DNA-seq, DNA sequencing; ELISA, enzyme-linked immunosorbent assay; FISH, fluorescence *in situ* hybridization; HCC, hepatocellular carcinoma; HGSOC, high-grade serous ovarian carcinoma; IHC, immunohistochemistry; LCT, Leydig cell tumor; NGS, next-generation sequencing; NM, not mentioned; NMIBC, non-muscle-invasive bladder cancer; No., number; PCR, polymerase chain reaction; RISH, RNA-*in situ* hybridization; RNA-seq, RNA sequencing; RT-qPCR, reverse transcription quantitative polymerase chain reaction; SDC, salivary duct carcinoma; USA, United States of America; WB, western blot; WES, whole-exome sequencing.

^a^Conference abstract only (no corresponding full-text publication available).

**Figure 2:**
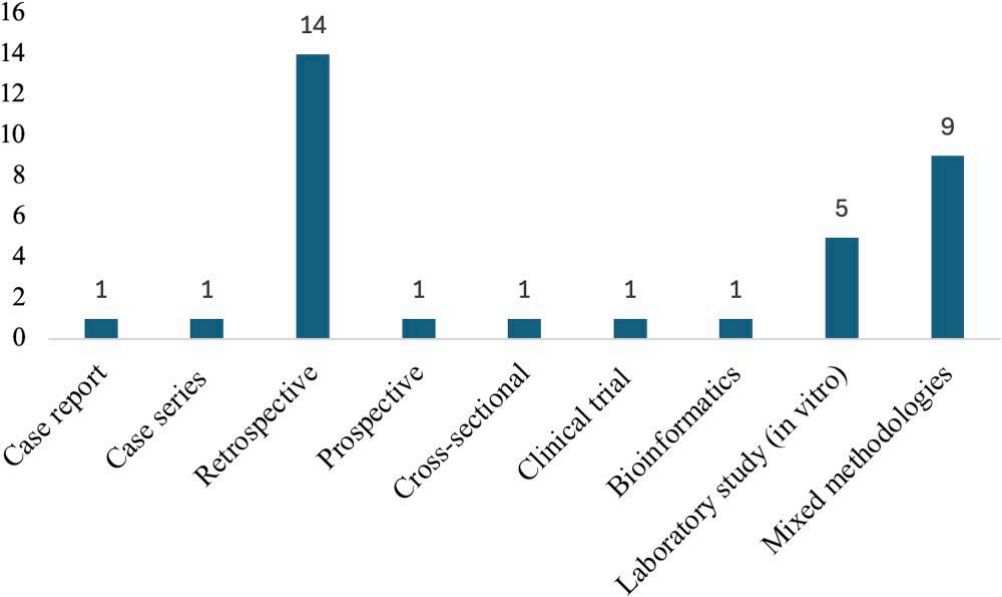
Distribution of the methodological designs of the included studies. Mixed-method studies were defined as studies combining an observational study design with *in vitro* and/or *in vivo* experimental components.

The included studies employed a wide range of methodological designs. The majority were retrospective cohort studies (*n* = 14) [13, 21–23, 29, 30, 32, 36–40, 43, 50], followed by nine studies that used mixed methods, combining observational methods with either *in vivo*, *in vitro*, or both [12, 20, 26, 27, 31, 35, 42, 46, 47]. Five studies were laboratory *in vitro* studies [19, 24, 25, 41, 44]. The remaining six studies included one clinical trial [34], one bioinformatic tool development study [48], one cross-sectional study [45], one prospective cohort study [33], one case series [49], and one case report [28]. Study designs are reported in Supplementary File S4, and a visual summary is provided in Fig. 2.

BrCa was the predominant focus of the included literature, with 16 of 34 (47.1%) studies evaluating BrCa exclusively [12, 21, 23, 24, 26, 27, 33, 34, 40–44, 46, 47, 50]. One study (2.9%) looked at BrCa and hepatocellular carcinoma (HCC) together [19]. Nine focused on SDC (26.5%) [13, 20, 22, 25, 28–30, 36, 38], and two focused on HCC [31, 35] (Supplementary Table S1).

### 3.4. Included clinical cases

Of the 4855 cases included, 4143 were female, and 196 were male; sex was not reported for the remaining 516 cases. Among 4855 patients, BrCa constituted the largest cohort (*n* = 4057; 83.6%). Lung adenocarcinoma was the second most common group (*n* = 515; 10.6%), followed by SDC (*n* 172; 3.5%). Less frequently reported cancers included HCC (*n* = 28; 0.6%), testicular Leydig cell tumor (LCT) (*n* = 27; 0.6%), ovarian LCT (*n* = 2), non-muscle-invasive bladder cancer (NMIBC) (*n* = 23; 0.5%), extra-mammary Paget’s disease (*n* = 18; 0.4%), high-grade serous ovarian cancer (HGSOC) (*n* = 11; 0.2%), and two cases with ovarian and uterine cancers that were not otherwise specified (Table 2).

**Table 2:** Summary of *AR-V7* and AR expression across clinical cases with non-prostatic cancers.

Cancer type	Total cases	Total *AR-V7* positive (%)	Total AR positive (%)^a^
Breast cancer	4057	43/177 by IHC, 228/2823 by PCR/GS-based techniques, and 544/1057 by splice-junction inference, giving 815/4057 (20.1%) in total.	66/138 by IHC and 334/741 by PCR/GS-based techniques, giving 400/879 (45.5%) in total.
Lung adenocarcinoma	515	0/515 (0%)	Not investigated
Salivary duct carcinoma	172	2/17 by IHC, 2/8 by RISH, 91/147 by PCR/GS-based techniques, giving 95/172 (55.2%) in total.	42/52 positive by IHC, 105/112 by PCR/GS-based techniques, giving 147/164 (89.6%) total.
Non-muscle-invasive bladder cancer	23	19/23 (82.6%)	15/23 (65.2%)
Hepatocellular carcinoma	28	16/28 (57.1%)	19/28 (67.9%)
Extra-mammary Paget’s disease	18	1/18	12/18
Testicular Leydig cell tumor	27	0/27 (0%)	12/27 (44.4%)
Ovarian Leydig cell tumor	2	0/2	0/2
High-grade serous ovarian cancer	11	0/11	Not investigated
Gynecologic cancers, not otherwise specified	2	2/2	2/2
Total	4855	948/4855 (19.5%)	607/1143 (53.1%)

AR, androgen receptor; *AR-V7*, androgen receptor splice variant 7; GS, generation sequencing; IHC, immunohistochemistry; PCR, polymerase chain reaction; RISH, RNA-*in situ* hybridization.

^a^AR status was available only for a subset of clinical cases; therefore, percentages are calculated using only cases with reported AR status.

### 3.5. Clinical detection methods of *AR-V7*

*AR-V7* detection methods varied considerably across studies and included both RNA-based assays [such as RNA sequencing, whole-transcriptome sequencing, RNA-*in situ* hybridization (RISH), and PCR-based assays] and protein-based assays. Two studies used RNA sequencing to detect *AR-V7* [39, 49], one combined DNA and RNA sequencing [34], and one used the OncoExTra assay, a tumor-normal whole-exome/whole-transcriptome sequencing platform [43]. Together, these studies comprised 1965 (40.5%) patients. PCR-based assays, IHC, and RISH were used less frequently [21, 28, 29, 33, 36, 38].

Several studies employed complementary detection strategies by combining RNA-based and protein-based assays [13, 26, 32, 37, 40, 50], while one study integrated RNA sequencing with anchored multiplex PCR and splice-junction analysis [48]. Detailed detection methods for each study are provided in Table 1, and a comprehensive summary is presented in Supplementary Table S2.

A notable methodological exception was the study by Hickey et al. [12], which analyzed 1057 BrCa cases from The Cancer Genome Atlas (TCGA) together with 54 prospectively collected clinical BrCa specimens. In the TCGA cohort, *AR-V7* expression was inferred from RNA sequencing–detected exon 3–cryptic exon 3 (CE3) splice-junction reads rather than being uniformly confirmed using *AR-V7* isoform-specific assays. In contrast, the prospectively collected clinical specimens were evaluated using isoform-specific methods, including PCR, western blotting, IHC, and dual-label immunofluorescence. Because transcript-level splice-junction inference differs fundamentally from direct isoform-specific detection, results from the TCGA cohort were evaluated separately in a sensitivity analysis of *AR-V7* prevalence.

### 3.6. Prevalence of *AR-V7* expression in clinical cases

Across the included clinical studies, *AR-V7* positivity was reported in 948 of 4855 (19.5%) clinical cases with non-prostatic cancers (Table 2). However, 544 of these positive cases originated from the TCGA BrCa cohort reported by Hickey et al. [12]. Among the remaining positive cases, 45 were detected by IHC, 2 by RISH, and 357 by PCR or other RNA-based assays.

To evaluate the influence of conference abstracts on the overall prevalence estimate, a sensitivity analysis was performed after excluding the six abstract-only studies [21, 23, 39, 43, 49, 50]. After exclusion, 853 of 2552 clinical cases were *AR-V7*-positive, corresponding to a prevalence of 33.4%, compared with the overall prevalence of 19.5% (948/4855). This increase was primarily driven by the exclusion of the large conference abstract by Gatalica et al. [43], which contributed 1944 cases, of which only 20 were *AR-V7*-positive. When this study was retained, and the remaining five abstract-only studies were excluded, the prevalence remained essentially unchanged (19.4%), indicating that the observed difference was largely attributable to this single influential cohort rather than to the inclusion of conference abstracts overall.

A second sensitivity analysis was performed after excluding the TCGA cohort reported by Hickey et al. [12]. Following exclusion, 404 of 3798 clinical cases were *AR-V7*-positive, corresponding to a prevalence of 10.6%, compared with the overall prevalence of 19.5% (948/4855).

### 3.7. Prevalence of *AR-V7* expression by tumor type

Tumor-specific *AR-V7* prevalence varied considerably across different cancers (Table 2). Within BrCa, 815 of 4057 patients (20.1%) were *AR-V7*-positive [12, 21, 23, 26, 32–34, 40, 42, 43, 46, 47, 50]. Notably, 544 of the *AR-V7*-positive cases originated from the TCGA cohort reported by Hickey et al. [12]. Excluding this cohort decreased *AR-V7* prevalence in BrCa to 9.0% (271/3000).

In contrast, *AR-V7* expression was detected in 95 of 172 SDC cases (55.2%) [13, 20, 22, 25, 28–30, 38]. Similarly, positivity was observed in 16 of 28 HCC cases (57.1%) [31] and in 19 of 23 NMIBCs (82.6%) [45]; however, these estimates were derived from relatively small cohorts. Interestingly, no *AR-V7* positivity was reported among 515 lung adenocarcinomas [48], 27 testicular LCTs [37], 2 ovarian LCTs [37], and 11 HGSOCs [39].

### 3.8. Prevalence of wild-type AR expression in clinical cases

Twenty-three studies comprising 1143 non-prostatic cancer cases investigated the expression of wild-type AR [12, 13, 20, 22, 23, 25, 26, 28, 30–34, 36–38, 40, 42, 45–47, 49, 50]. Three studies, including 190 cases, assessed AR expression exclusively by IHC, of which 108 (56.8%) were positive [20, 23, 38]. The remaining 20 studies employed molecular or genomic-based assays (e.g. PCR, RNA sequencing) and evaluated 953 cases, identifying AR expression in 499 (52.4%) [12, 13, 22, 25, 26, 28, 30–34, 36, 37, 40, 42, 45–47, 49, 50].

Among the 4057 BrCa cases included, AR status was specifically investigated in 879 [12, 23, 26, 32–34, 40, 42, 46, 47, 50]. Of these, 66 (7.5%) were positive by IHC [23], while 334 (38.0%) were positive using molecular confirmatory methods [12, 26, 32–34, 40, 42, 46, 47, 50]. In SDC, AR was examined in 164 of 172 cases [13, 20, 22, 25, 28, 30, 36, 38], with 42 (25.6%) positive by IHC [20, 38] and 105 (64.0%) positive by molecular methods [13, 22, 25, 28, 30, 36]. In NMIBC, AR expression was detected in 15 of 23 (65.2%) cases [45]. Similarly, 19 of 28 (67.9%) HCC cases were positive [31]. Expression was also reported in 12 of 18 (66.7%) individuals with extra-mammary Paget’s disease [32, 49]. Detailed distribution of AR expression across cancer types is in Table 2.

### 3.9. *AR-V7* expression by treatment status

*AR-V7* was expressed in both treatment-naive and previously treated patients in several non-prostatic cancers. In SDC, multiple studies reported baseline *AR-V7* expression in treatment-naive patients, with reported prevalence estimates of 37% (13/35) [20], 48% (13/27) [13], and 95.3% (41/43) [36].

In treatment-naive BrCa cohorts, reported *AR-V7* prevalence ranged from 1% to 68.4%, including, 24.3% (43/177) [21], 68.4% (52/76) [46], 53.7% (29/54) [12], 19.5% (8/41) in CTCs [33], and 1% (20/1944) in a large clinical cohort [43]. Moreover, *AR-V7* was reported in apocrine ductal carcinoma *in situ* (DCIS) [32, 43, 50]. *AR-V7* was also detected in BrCa cases with prior systemic therapy. In a study by Aceto et al. [26], CTCs from 22 patients with metastatic BrCa who underwent multiple lines of therapy, including aromatase inhibitors, were analyzed for *AR-V7,* which was detected in 9 of them. The authors further reported that AR expression in CTCs correlated with the duration of aromatase inhibitor therapy and suggested a potential association with acquired endocrine resistance [26]. In another study of AR-positive metastatic triple-negative BrCa, *AR-V7* expression increased following treatment with AR antagonists in two patients [34].

Additionally, Sarıkaya et al. [45] reported *AR-V7* mRNA expression in 19/23 patients with NMIBC and no prior exposure to Bacillus Calmette–Guérin or intravesical treatment. On the other hand, *AR-V7* was not detected in the 11 chemo-naive HGSOC specimens [39].

### 3.10. Treatment outcomes

Clinical outcome data were limited and heterogeneous across the included studies. Patients with SDC who had detectable *AR-V7* in pretreatment samples often did not respond to subsequent ADT with agents such as leuprorelin and bicalutamide [13, 29, 36]. Further supporting this, one case with markedly elevated pretreatment *AR-V7* levels in their CTCs experienced rapid disease progression on abiraterone [28].

Similarly, in BrCa, six patients with wild-type AR (three *AR-V7*-positive and three *AR-V7*-negative) received anti-androgen therapy; however, treatment was discontinued in all patients due to disease progression [40]. Another study reported that patients with *AR-V7*-positive CTCs were associated with earlier relapse and reduced overall survival [33]. Conversely, one patient with AR-positive but *AR-V7*-negative apocrine DCIS responded to anti-androgen therapy [50].

### 3.11. Preclinical and mechanistic evidence

Twelve of 34 studies examined cancer cell lines [12, 19, 20, 24, 27, 31, 35, 41, 42, 44, 46, 47] (Table 1 and Supplementary Table S3). Together, these 12 papers examined 60 cell lines, 33 of which were unique. Of the 60 cell lines identified, 46 were BrCa, 12 were HCC, one was SDC, and one was a salivary epidermoid carcinoma cell line. Prevalence of *AR-V7* and wild-type AR expression across the investigated cell lines is presented in Table 1 and Supplementary Tables S4 and S5.

Several studies further investigated the biological role and regulation of *AR-V7* expression in preclinical models. In BrCa cell lines, androgen deprivation increased the expression of multiple AR splice variants, including *AR-V7* [12, 19, 27, 46]. In HCC cell lines, HCCLM3 and SNU-475 exhibited ligand-independent *AR-V7* signaling that remained resistant to enzalutamide, whereas SNU-423 demonstrated ligand-dependent AR activation that was sensitive to enzalutamide [31]. Additional mechanistic findings, including studies evaluating signaling pathways, AR splice-variant regulation, and therapeutic interventions, are summarized in Supplementary Table S3.

### 3.12. Prevalence and co-expression of *AR-V7* and AR in BrCa cell lines

Nine studies reported a total of 46 BrCa cell lines, of which 22 were unique and included in the analysis [12, 19, 24, 27, 41, 42, 44, 46, 47]. Among the 22 unique BrCa cell lines, *AR-V7* expression was detected in 13, while AR expression was observed in 14 (Supplementary Table S5).

*AR-V7* positivity was identified across multiple molecular subtypes, including ER-positive luminal models (BT474, MCF7, T47D, ZR75-1, and the tamoxifen-resistant MCF7 derivative LLC2), HER2-enriched cell lines (HCC202, HCC1419, MDA-MB-453, MDA-MB-361, UACC-893), and AR-driven triple-negative cell line models (CAL-148, MDA-MB-231, MFM223).

AR expression showed an almost identical distribution to *AR-V7*. *AR-V7* was not detected in any cell line lacking AR expression, whereas AR positivity did not always coincide with *AR-V7* detection. For example, CAL-51 was the only model that expressed AR but lacked *AR-V7*.

Across *AR-V7*-positive models, multiple additional AR splice variants were also reported, including *AR-V1*, *AR-V3*, *AR-V4*, *AR-V9*, *AR-V45*, *AR-V15*, *AR-V16*, *AR-V17*, *AR-V18*, and *AR-V567es*, indicating the presence of diverse AR splice variants within these models.

## 4. Discussion

In this systematic review, *AR-V7* was identified in 948 of 4855 clinical cases (19.5%) across multiple non-prostatic cancers. This overall proportion provides an overview of the currently available evidence rather than a single prevalence estimate applicable to all non-prostatic cancers, as the included studies differed substantially in tumor type, study design, and *AR-V7* detection methods. BrCa accounted for the majority of both included clinical cases (4057/4855; 83.6%) and *AR-V7*-positive tumors (815/948, 86.0%), reflecting the much larger body of available literature for this malignancy [12, 21, 23, 26, 32–34, 40, 42, 43, 46, 47, 50]. Consequently, the overall proportion is influenced predominantly by BrCa-specific studies. Although higher tumor-specific proportions were reported in SDC, HCC, and NMIBC, these estimates were derived from substantially smaller cohorts and should therefore be interpreted cautiously.

The sensitivity analyses highlighted two distinct sources of variability in the reported prevalence estimates. First, the conference abstract sensitivity analysis demonstrated the influence of exceptionally large cohorts on the overall prevalence estimate. The marked increase in prevalence following exclusion of conference abstracts was driven almost entirely by the large BrCa cohort reported by Gatalica et al. [43], indicating that this finding reflected the impact of a single low-prevalence study rather than the publication format itself.

The second sensitivity analysis further underscored the influence of detection methodology on the reported prevalence estimates. When analyses were restricted to studies employing isoform-specific PCR, RNA-based, or protein-based methods, *AR-V7* positivity in BrCa remained low (9%), suggesting that the higher prevalence observed in the primary analysis was largely attributable to methodological differences rather than a uniformly high prevalence across BrCa. These findings highlight the importance of interpreting prevalence estimates within the context of the assay used and support the need for greater standardization of *AR-V7* detection methods in future studies.

Beyond the variability in the reported prevalence estimates, the strength of evidence differed considerably across tumor types. Although relatively high *AR-V7* positivity was observed in SDC (55.2%) [13, 20, 22, 25, 28–30, 38], HCC (57.1%) [31], and NMIBC (82.6%) [45], these findings should not be interpreted equivalently, as the quantity and quality of the available evidence varied substantially between malignancies. BrCa currently provides the strongest evidence for *AR-V7* detectability because it has been investigated across the largest number of patients and independent cohorts. However, confidence in its clinical predictive value remains limited because many available studies were observational, substantial assay heterogeneity was present, and the overall risk of bias was frequently moderate to high. In contrast, evidence for SDC is of moderate strength. Despite the relatively small overall sample size (*n* = 172), multiple independent studies consistently demonstrated *AR-V7* expression, together with biologic plausibility and preliminary clinical associations with response to ADT [13, 20, 22, 25, 28–30, 38]. Nevertheless, the available evidence remains insufficient to establish a reliable predictive role because clinical outcome data are limited and are derived primarily from observational studies.

Evidence for HCC and NMIBC remains preliminary. Although both tumor types demonstrated relatively high reported *AR-V7* positivity, these estimates were derived from very small cohorts and therefore require validation in larger clinical studies before meaningful conclusions regarding prevalence or clinical significance can be drawn. Accordingly, confidence in the current evidence for these tumor types remains low. Conversely, no *AR-V7* expression was detected in the relatively large lung adenocarcinoma cohort, suggesting that *AR-V7* is unlikely to represent a universal feature of non-prostatic cancers and may instead exhibit tumor-specific expression patterns [48]. For gynecologic tumors, extra-mammary Paget’s disease, and LCTs, the currently available evidence remains insufficient because conclusions are based on isolated studies or very limited patient numbers [37, 39, 43, 49].

Importantly, this review highlights a difference in the temporal pattern of *AR-V7* expression between PCa and several non-prostatic cancers. In PCa, *AR-V7* is classically regarded as a therapy-induced alteration, emerging under selective pressure from androgen deprivation or next-generation AR-signaling inhibitors [4, 51]. However, a recent study identified *AR-V7* in a small subset of patients with treatment-naive primary PCa (∼6/43 patients) using ultra-deep RNA sequencing [52]. Interestingly, *AR-V9* was found in 20% of cases, and *AR-45* in 41% [52].

In contrast, our review identified *AR-V7* expression in treatment-naive tumors across several non-prostatic malignancies. High baseline *AR-V7* prevalence was consistently reported in untreated SDC [13, 20, 25, 36] and BrCa subtypes, including luminal, HER2-enriched, and triple-negative cases [12, 21, 33, 43, 46]. Similar expression patterns were observed in HCC [31, 35] and NMIBC [45]. On the other hand, HGSOC showed no *AR-V7* expression in chemo-naive specimens [39]. This might suggest that, in at least some non-prostatic cancers, *AR-V7* expression may be a preexisting feature rather than a solely therapy-emergent alteration. Several studies also reported an increase in *AR-V7* expression following treatment in BrCa [12, 26, 27, 34, 42, 46]. Taken together, these observations suggest that *AR-V7* may function as both an intrinsic and adaptive resistance mechanism in selected non-prostatic cancers. The detection of *AR-V7* before treatment supports the possibility that preexisting *AR-V7*-positive clones are present in at least a subset of tumors, while subsequent therapy may further enrich these clones rather than initiating variant formation *de novo*.

This distinction challenges the prevailing PCa–centric paradigm of *AR-V7* biology. It suggests that, in non-prostatic cancers, particularly BrCa, *AR-V7* may represent a preexisting molecular state that predisposes tumors to primary resistance to AR-targeted therapies. Specifically, in apocrine carcinomas of the breast, *AR-V7* may be seen in their precursor lesions (apocrine DCIS), suggesting an early event in tumorigenesis [43, 50]. Therapeutic exposure may further enrich for *AR-V7-*positive clones, but the presence of *AR-V7* before treatment initiation highlights fundamental differences in AR regulation between prostatic and non-prostatic cancers.

From a clinical perspective, these findings suggest that *AR-V7* may represent a candidate biomarker worthy of further investigation in selected non-prostatic cancers. However, substantial heterogeneity in *AR-V7* detection methods, biological specimens, and sampling time points currently limits clinical translation. Harmonization of *AR-V7* assays, standardized reporting, and incorporation of *AR-V7* testing into prospective, biomarker-driven clinical trials will be essential before routine implementation can be recommended.

Across all BrCa cell lines, *AR-V7* expression was detected only in AR-positive models. None of the included studies reported *AR-V7* expression in a BrCa cell line lacking wild-type AR, suggesting that expression of the full-length AR transcript may be a prerequisite for splice-variant generation in these experimental models [19, 27, 44]. However, because AR status was not systematically evaluated across all clinical cohorts or tumor types, these findings should not be generalized beyond the available preclinical evidence. *AR-V7-*positive cell lines also commonly co-expressed additional AR splice variants, reflecting complex posttranscriptional regulation that may contribute to phenotypic heterogeneity and drug resistance [24, 46].

The strengths of this review include its comprehensive scope, the inclusion of both clinical and preclinical evidence, and the systematic evaluation of *AR-V7* across a wide range of non-prostatic cancers. In addition, by separately examining treatment-naive and posttreatment settings and exploring the influence of different detection methods, this review provides a comprehensive overview of the current evidence and identifies important areas requiring further investigation.

Nevertheless, several limitations must be acknowledged. The included studies were heterogeneous in design, *AR-V7* detection methodology, and outcome reporting, precluding quantitative meta-analysis. Many studies were descriptive, observational, or preclinical, and the overall risk of bias was moderate to high in a substantial proportion. Additionally, data for several tumor types were limited to small cohorts or single studies, restricting the generalizability of prevalence estimates. The differences in *AR-V7* assays, including the specificity of antibodies and other detection platforms, may have contributed to variability in *AR-V7* detection across studies and should be considered when interpreting the reported findings. These limitations highlight the need for well-designed prospective studies with standardized *AR-V7* assessment. Finally, some included studies involved overlapping authors/institutions; despite independent assessments, residual cohort overlap and a potential intellectual bias cannot be fully excluded.

## 5. Conclusion

*AR-V7* can be detected across multiple non-prostatic cancers, although its reported prevalence varies substantially by tumor type and detection method. Unlike the classical paradigm in PCa, *AR-V7* was identified in treatment-naive tumors across several non-prostatic cancers, suggesting that it may represent a preexisting molecular feature in at least a subset of cancers. In selected malignancies, like apocrine carcinoma of the breast, *AR-V7* was also detected in precursor, noninvasive (*in situ*) lesions, further supporting its biological relevance in tumor development. Together, these findings expand the current understanding of AR signaling beyond PCa and support *AR-V7* as a potential candidate biomarker of intrinsic or adaptive resistance that requires further clinical validation. Standardized assays and prospective clinical validation are required before routine clinical implementation.

## Supplementary Material

bgag052_Supplementary_Data

## Data Availability

Data used for all analyses are provided in the *Supplementary Materials* associated with this article.

## References

[1] Chang C, Lee SO, Wang RS et al Androgen receptor (AR) physiological roles in male and female reproductive systems: lessons learned from AR-knockout mice lacking AR in selective cells. Biol Reprod 2013;89:21. 10.1095/biolreprod.113.10913223782840 PMC4076350

[2] Walters KA, Simanainen U, Handelsman DJ. Molecular insights into androgen actions in male and female reproductive function from androgen receptor knockout models. Hum Reprod Update 2010;16:543–58. 10.1093/humupd/dmq003 20231167

[3] Aurilio G, Cimadamore A, Mazzucchelli R et al Androgen receptor signaling pathway in prostate cancer: from genetics to clinical applications. Cells 2020;9:2653. 10.3390/cells912265333321757 PMC7763510

[4] Kirby M, Hirst C, Crawford ED. Characterising the castration-resistant prostate cancer population: a systematic review. Int J Clin Pract 2011;65:1180–92. 10.1111/j.1742-1241.2011.02799.x 21995694

[5] Young FP, Becker TM, Nimir M et al Biomarkers of castrate resistance in prostate cancer: androgen receptor amplification and T877A mutation detection by Multiplex droplet digital PCR. J Clin Med 2022;11:257. 10.3390/jcm1101025735011998 PMC8745706

[6] Hörnberg E, Ylitalo EB, Crnalic S et al Expression of androgen receptor splice variants in prostate cancer bone metastases is associated with castration-resistance and short survival. PLoS One 2011;6:e19059. 10.1371/journal.pone.001905921552559 PMC3084247

[7] Antonarakis ES, Lu C, Wang H et al AR-V7 and resistance to enzalutamide and abiraterone in prostate cancer. N Engl J Med 2014;371:1028–38. 10.1056/NEJMoa131581525184630 PMC4201502

[8] Antonarakis ES, Lu C, Luber B et al Androgen receptor splice variant 7 and efficacy of taxane chemotherapy in patients with metastatic castration-resistant prostate cancer. JAMA Oncol 2015;1:582–91. 10.1001/jamaoncol.2015.134126181238 PMC4537351

[9] Barbier RH, Chau CH, Figg WD. Androgen receptor splice variant 7 (AR-V7) and AR full-length (AR-FL) as predictive biomarkers of therapeutic resistance: partners in crime? BJU Int 2019;124:549–50. 10.1111/bju.1486931908596 PMC6943979

[10] Dehm SM, Schmidt LJ, Heemers HV et al Splicing of a novel androgen receptor exon generates a constitutively active androgen receptor that mediates prostate cancer therapy resistance. Cancer Res 2008;68:5469–77. 10.1158/0008-5472.Can-08-059418593950 PMC2663383

[11] Nicolò E, Reduzzi C, Pierga J-Y et al International expert consensus on the clinical integration of circulating tumor cells in solid tumors. Eur J Cancer 2025;231:116050. 10.1016/j.ejca.2025.116050 41172567

[12] Hickey TE, Irvine CM, Dvinge H et al Expression of androgen receptor splice variants in clinical breast cancers. Oncotarget 2015;6:44728–44. 10.18632/oncotarget.629626554309 PMC4792588

[13] Gargano SM, Senarathne W, Feldman R et al Novel therapeutic targets in salivary duct carcinoma uncovered by comprehensive molecular profiling. Cancer Med 2019;8:7322–9. 10.1002/cam4.260231609094 PMC6885888

[14] Munn Z, Barker TH, Moola S et al Methodological quality of case series studies: an introduction to the JBI critical appraisal tool. JBI Evid Synth 2020;18:2127–33. 10.11124/jbisrir-d-19-00099 33038125

[15] Barker TH, Hasanoff S, Aromataris E et al The revised JBI critical appraisal tool for the assessment of risk of bias for analytical cross-sectional studies. JBI Evid Synth 2026;24:401–8. 10.11124/jbies-24-00523 40521701

[16] Sterne JAC, Savović J, Page MJ et al Rob 2: a revised tool for assessing risk of bias in randomised trials. Br Med J 2019;366:l4898. 10.1136/bmj.l4898 31462531

[17] Pamies D, Leist M, Coecke S et al Guidance document on good cell and tissue culture practice 2.0 (GCCP 2.0). Altex 2022;39:30–70. 10.14573/altex.2111011 34882777

[18] Almeida JL, Cole KD, Plant AL. Standards for cell line authentication and beyond. PLoS Biol 2016;14:e1002476. 10.1371/journal.pbio.100247627300367 PMC4907466

[19] Hu DG, Hickey TE, Irvine C et al Identification of androgen receptor splice variant transcripts in breast cancer cell lines and human tissues. Horm Cancer 2014;5:61–71. 10.1007/s12672-014-0171-424570075 PMC10358119

[20] Mitani Y, Rao PH, Maity SN et al Alterations associated with androgen receptor gene activation in salivary duct carcinoma of both sexes: potential therapeutic ramifications. Clin Cancer Res 2014;20:6570–81. 10.1158/1078-0432.Ccr-14-174625316813 PMC4268116

[21] Asano Y, Kashiwagi S, Goto W et al Abstract 3939: clinical significance of expression of androgen-receptor splice variant-7 (AR-V7) in neoadjuvant chemotherapy for breast cancer. Cancer Res 2016;76:3939–3939. 10.1158/1538-7445.Am2016-3939

[22] Dalin MG, Desrichard A, Katabi N et al Comprehensive molecular characterization of salivary duct carcinoma reveals actionable targets and similarity to apocrine breast cancer. Clin Cancer Res 2016;22:4623–33. 10.1158/1078-0432.Ccr-16-063727103403 PMC5026550

[23] Singh B, Deng F-M, Kane Y et al Abstract P3-05-08: high prevalence of splicing variant AR-V7 in triple negative breast carcinoma. Cancer Res 2016;76:P3-05-08–P3-05-08. 10.1158/1538-7445.Sabcs15-p3-05-08

[24] Stossi F, Dandekar RD, Bolt MJ et al High throughput microscopy identifies bisphenol AP, a bisphenol A analog, as a novel AR down-regulator. Oncotarget 2016;7:16962–74. 10.18632/oncotarget.765526918604 PMC4941363

[25] Li J, Mitani Y, Rao PH et al Establishment and genomic characterization of primary salivary duct carcinoma cell line. Oral Oncol 2017;69:108–14. 10.1016/j.oraloncology.2017.04.00728559013 PMC5531198

[26] Aceto N, Bardia A, Wittner BS et al AR expression in breast cancer CTCs associates with bone metastases. Mol Cancer Res 2018;16:720–7. 10.1158/1541-7786.Mcr-17-048029453314 PMC5882540

[27] Braga DL, Mota STS, Zóia MAP et al Ethanolic extracts from *Azadirachta indica* leaves modulate transcriptional levels of hormone receptor variant in breast cancer cell lines. Int J Mol Sci 2018;19:1879. 10.3390/ijms1907187929949923 PMC6073126

[28] Cappelletti V, Miodini P, Reduzzi C et al Tailoring treatment of salivary duct carcinoma (SDC) by liquid biopsy: ARv7 expression in circulating tumor cells. Ann Oncol 2018;29:1599–601. 10.1093/annonc/mdy141 32138836

[29] Kang H, Antonarakis ES, Luo J et al Detection of AR-V7 transcript with RNA in situ hybridization in human salivary duct cancer. Oral Oncol 2018;84:134–6. 10.1016/j.oraloncology.2018.06.026 30122219

[30] Yang RK, Zhao P, Lu C et al Expression pattern of androgen receptor and AR-V7 in androgen-deprivation therapy-naïve salivary duct carcinomas. Hum Pathol 2019;84:173–82. 10.1016/j.humpath.2018.09.00930267779 PMC7098468

[31] Dauki AM, Blachly JS, Kautto EA et al Transcriptionally active androgen receptor splice variants promote hepatocellular carcinoma progression. Cancer Res 2020;80:561–75. 10.1158/0008-5472.Can-19-111731685543 PMC7002251

[32] Gatalica Z, Vranic S, Krušlin B et al Comparison of the biomarkers for targeted therapies in primary extra-mammary and mammary Paget's disease. Cancer Med 2020;9:1441–50. 10.1002/cam4.282031899853 PMC7013075

[33] Kasimir-Bauer S, Keup C, Hoffmann O et al Circulating tumor cells expressing the prostate specific membrane antigen (PSMA) indicate worse outcome in primary, non-metastatic triple-negative breast cancer. Front Oncol 2020;10:1658. 10.3389/fonc.2020.0165833014830 PMC7497312

[34] Lehmann BD, Abramson VG, Sanders ME et al TBCRC 032 IB/II multicenter study: molecular insights to AR antagonist and PI3K inhibitor efficacy in patients with AR(+) metastatic triple-negative breast cancer. Clin Cancer Res 2020;26:2111–23. 10.1158/1078-0432.Ccr-19-217031822498 PMC7196503

[35] Song H, Sun N, Lin L et al Splicing factor PRPF6 upregulates oncogenic androgen receptor signaling pathway in hepatocellular carcinoma. Cancer Sci 2020;111:3665–78. 10.1111/cas.1459532745318 PMC7540998

[36] van Boxtel W, Verhaegh GW, van Engen-van Grunsven IA et al Prediction of clinical benefit from androgen deprivation therapy in salivary duct carcinoma patients. Int J Cancer 2020;146:3196–206. 10.1002/ijc.3279531745978 PMC7187215

[37] Kruslin B, Gatalica Z, Hes O et al TERT gene fusions characterize a subset of metastatic Leydig cell tumors. Clin Genitourin Cancer 2021;19:333–8. 10.1016/j.clgc.2021.02.00233741265 PMC9907364

[38] Schvartsman G, Bell D, Rubin ML et al The tumor immune contexture of salivary duct carcinoma. Head Neck 2021;43:1213–9. 10.1002/hed.26587 33576119

[39] Wilson B, Xiu J, Ulm M et al Expression of androgen receptor splice variant, AR-V7, in high-grade serous ovarian cancer. Gynecol Oncol 2021;162:S157–8. 10.1016/S0090-8258(21)00941-0

[40] Ferguson DC, Mata DA, Tay TK et al Androgen receptor splice variant-7 in breast cancer: clinical and pathologic correlations. Mod Pathol 2022;35:396–402. 10.1038/s41379-021-00924-534593966 PMC8863633

[41] Ali B, El-Abhar H, Abdallah D et al The enzalutamide and EPI-001 modulate cell proliferation and metastasis markers in T47D by targeting AR/ARV7. Indones J Pharm 2023;34:617–29. 10.22146/ijp.7416

[42] Asemota S, Effah W, Young KL et al Identification of a targetable JAK-STAT enriched androgen receptor and androgen receptor splice variant positive triple-negative breast cancer subtype. Cell Rep 2023;42:113461. 10.1016/j.celrep.2023.11346137979170 PMC10872270

[43] Gatalica Z, Vranic S, Hall D et al Androgen receptor splice variant-7 is an early pathogenic event in breast carcinomas with apocrine differentiation. Lab Invest 2024;104:S160–1. 10.1016/j.labinv.2024.100428

[44] Kuroiwa Y, Ito K, Nakayama J et al Analysis of the responsiveness to antiandrogens in multiple breast cancer cell lines. Genes Cells 2024;29:301–15. 10.1111/gtc.13105 38366725

[45] Sarıkaya EA, Korhan P, Incir C et al Evaluation of androgen receptor and androgen receptor splice-variant 7 in bladder cancer; a novel approach into an ancient topic. World J Urol 2024;42:459. 10.1007/s00345-024-05166-z39083104 PMC11291539

[46] Srivastava TP, Ajmeriya S, Goel I et al Prognostic role of androgen receptor splice variant 7 (AR-V7) in the pathogenesis of breast cancer. BMC Cancer 2024;24:1398. 10.1186/s12885-024-13165-x39538154 PMC11562864

[47] Ali BM, El-Abhar HS, Mohamed G et al A study of the role of androgen receptor and androgen receptor variant 7 in TNBC patients and the effect of their targeting by enzalutamide and EPI-001 in MDA-MB-231. J Steroid Biochem Mol Biol 2025;245:106636. 10.1016/j.jsbmb.2024.106636 39536950

[48] Bian W, Zhang B, Song Z et al SplitFusion enables ultrasensitive gene fusion detection and reveals fusion variant-associated tumor heterogeneity. Patterns (N Y) 2025;6:101174. 10.1016/j.patter.2025.10117440041857 PMC11873004

[49] Wang L, Vasudevaraja V, Tran I et al Novel androgen receptor splice variant 7 in gynecologic tumors. Int J Gynecol Pathol 2025;44:88–93. 10.1097/pgp.0000000000001029 38833720

[50] Gatalica Z, Ye J, Chen Y-Y et al Uncovering AR-v7 as a common early event in apocrine breast tumors: rethinking antiandrogen therapy strategies. Arch Pathol Lab Med 2025;149:e238–9.

[51] Sharp A, Coleman I, Yuan W et al Androgen receptor splice variant-7 expression emerges with castration resistance in prostate cancer. J Clin Invest 2019;129:192–208. 10.1172/jci12281930334814 PMC6307949

[52] Konieczkowski DJ, Otani K, Guan Z et al Androgen receptor splice variant expression and prostate cancer recurrence after salvage therapy. NPJ Precis Oncol 2025;10:4. 10.1038/s41698-025-01201-341326770 PMC12775408

