# Supplementary material for: The prevalence of the AR-V7 variant and its association with clinicopathologic characteristics in non-prostatic cancers—a systematic review
Source: Carcinogenesis. 2026 Aug 4;47(3):bgag052. doi: 10.1093/carcin/bgag052 (PMC13475121; doi:10.1093/carcin/bgag052)
Supplement: Supplementary file 1 [file bgag052_supplementary_data.zip › Highlights, revised.docx]

**Highlights**

- Androgen receptor splice variant 7 (*AR-V7*) is a well-established mediator of resistance to androgen receptor (AR) targeting therapies in prostate cancer (PCa).
- The prevalence, biological significance, and clinical relevance of *AR-V7* in non-prostatic cancers remain poorly defined.
- *AR-V7* is detectable across several non-prostatic cancers and may represent an intrinsic tumor-specific feature associated with resistance to AR-targeted therapy.
- Standardized assays and prospective studies are needed to define its predictive and prognostic value.
