# Supplementary material for: The prevalence of the AR-V7 variant and its association with clinicopathologic characteristics in non-prostatic cancers—a systematic review
Source: Carcinogenesis. 2026 Aug 4;47(3):bgag052. doi: 10.1093/carcin/bgag052 (PMC13475121; doi:10.1093/carcin/bgag052)
Supplement: Supplementary file 1 [file bgag052_supplementary_data.zip › New Supplementary Tables.docx]

**Supplementary Table 1: Overview of total papers investigating *AR-V7* expression in non-prostatic cancers**

| **Cancer Type** | **Total papers** | **References** |
| --- | --- | --- |
| Breast cancer and Hepatocellular carcinoma | 1 | (1) |
| Breast cancer | 16 | (2-17) |
| Lung adenocarcinoma | 1 | (18) |
| Salivary duct carcinoma | 9 | (19-27) |
| Non-muscle invasive bladder cancer | 1 | (28) |
| Hepatocellular carcinoma | 2 | (29, 30) |
| Extra-mammary and Mammary Paget's disease | 1 | (31) |
| Leydig cell tumor | 1 | (32) |
| High grade serous ovarian cancer | 1 | (33) |
| Gynecologic cancers, not otherwise specified | 1 | (34) |

**Supplementary Table 2: Summary of *AR-V7* detection methods across included studies.**

| **Detection method** | **Studies using this method** | **Total number of studies** | **Total number of patients** | **Total number of cell lines** |
| --- | --- | --- | --- | --- |
| ELISA + Immunocytostaining + WB | (11) | 1 | 0 | 1 |
| ELISA + IHC + bioinformatics analysis of RNA status | (16) | 1 | 251 | 1 |
| FISH + DNA sequencing + IHC + RT-qPCR + WB | (5, 19) | 2 | 35 | 5 |
| RNA sequencing | (33, 34) | 2 | 13 | 0 |
| RNA sequencing + DNA sequencing | (9) | 1 | 8 | 0 |
| OncoExTra test (a tumor-normal, whole-exome, whole-transcriptome assay) | (13) | 1 | 1944 | 0 |
| RNA-based sequencing + IHC | (6, 10, 17, 24, 31, 32) | 6 | 333 | 0 |
| SplitFusion algorithm on RNA sequencing + Anchored Multiplex PCR | (18) | 1 | 515 | 0 |
| RT-qPCR + IHC + WES + RNA sequencing | (20) | 1 | 16 | 0 |
| RT-qPCR + IHC + WES | (21) | 1 | 1 | 0 |
| RNA sequencing + IHC + RT-qPCR + WB | (30) | 1 | 0 | 5 |
| RNA sequencing, RT-qPCR, WB, WGS, immunofluorescence | (29) | 1 | 28 | 6 |
| RNA sequencing + IHC + WB + RT-qPCR + dual label immunofluorescence | (2) | 1 | 1111 | 8 |
| IHC | (3, 27) | 2 | 194 | 0 |
| IHC + RT-qPCR | (4) | 1 | 138 | 0 |
| IHC + RT-qPCR + WB | (12, 15) | 2 | 128 | 7 |
| immunofluorescent staining + RT-qPCR + WB | (14) | 1 | 0 | 18 |
| RT-qPCR | (1, 7, 8, 22, 26) | 5 | 86 | 9 |
| RT-qPCR + WB | (28) | 1 | 23 | 0 |
| RNA in situ hybridization | (23) | 1 | 8 | 0 |
| RNA in situ hybridization + IHC + RT-qPCR | (25) | 1 | 23 | 0 |

**ELISA:** Enzyme-linked immunosorbent assay; **FISH:** Fluorescence in situ hybridization; **IHC:** Immunohistochemistry; **PCR:** Polymerase chain reaction; **RT-qPCR:** Reverse transcription quantitative polymerase chain reaction; **WB:** Western blot; **WES:** Whole exome sequencing; **WGS:** Whole genome sequencing

**Supplementary Table 3: Summary of included in vitro cell line studies and their reported AR-related outcomes**

| **No.** | **Author, year** | **Included cell lines** | **Experimental results** |
| --- | --- | --- | --- |
| 1 | Mitani, 2014 (19) | RET981, A253 | - AR exon-1 knockdown suppressed RET981 growth more effectively than LBD targeting. - RET981 showed androgen-independent growth, whereas A253 did not respond to AR knockdown. |
| 2 | Dauki, 2020 (29) | HepG2, PLC/PRF/5, SNU-423, HCCLM3, HepG2/C3A, SNU-475 | - SNU-423 showed ligand-dependent, enzalutamide-sensitive AR signaling. - HCCLM3 and SNU-475 exhibited ligand-independent *AR-V7*-driven, enzalutamide-resistant signaling. - Both AR pathways promoted migration, invasion, and EMT. |
| 3 | Song, 2020 (30) | HCCLM3, PLC5, Huh7, HepG2, SMMC-7721 | - PRPF6 increased AR and *AR-V7* activity independently of DHT through AR self-transcription. |
| 4 | Hu, 2014 (1) | MFM223, MDA-MB-453, MDA-MB-231, ZR75.1, MCF7, T47D, HepG2 | - Androgen depletion enhanced the expression of AR-Vs (V1, V3, V7, V9) in MDA-MB-453 cells, an effect that was completely reversed by DHT treatment. |
| 5 | Hickey, 2015 (2) | MFM-223, MDA-MB-453, ZR-75-1, T47D, MDA-MB-231, MCF7, CAL-51, CAL-148 | - *AR-V7* promoted ligand-independent signaling and enzalutamide resistance in ER-negative breast cancer cells. |
| 6 | Stossi, 2016 (5) | MCF7, LLC2, T47D | - DHT induced AR nuclear translocation. - Enzalutamide blocked DHT-induced AR stabilization and nuclear localization. |
| 7 | Braga, 2018 (7) | MCF7 and MDA-MB-231 | - Neem leaf extract inhibited TNBC cell proliferation but increased *AR-V7* expression in MDA-MB-231 cells. |
| 8 | Ali, 2023 (11) | T47D | - EPI-001 and Enzalutamide inhibited proliferation by inducing S-phase arrest and downregulating key cyclins and NF-ĸB. |
| 9 | Asemota, 2023 (12) | MDA-MB-453, MDA-MB-231, BT549, MFM223 | - *AR-V7* drives aggressive breast cancer via JAK-STAT signaling, inhibited by UT-105 and ruxolitinib. |
| 10 | Kuroiwa, 2024 (14) | MDA-MB-157, MDA-MB-231, MDA-MB-468, HCC38, Hs578T, BT-549, MDA-MB-453, UACC-893, HCC202, SK-BR-3, HCC1954, HCC1419, MDA-MB-361, UACC-812, BT-474, ZR-75-1, T47D, MCF7 | - AR antagonists inhibited breast cancer proliferation via AR-independent RAF/MEK/ERK signaling; AR and *AR-V7* expression did not correlate with drug sensitivity. |
| 11 | Srivastava, 2024 (15) | MDA-MB-231, MCF7, MDA-MB-453 | - Bicalutamide and enzalutamide decreased AR-FL expression but increased *AR-V7* levels. |
| 12 | Ali, 2025 (16) | MDA-MB-231 | - EPI-001 and enzalutamide inhibited migration and reduced EMT markers without altering receptor protein levels. |

**AR:** Androgen receptor; **AR-FL:** Full-length Androgen Receptor; ***AR-V7*:** Androgen Receptor Variant 7; ***AR-Vs:*** Androgen Receptor Variants; **DHT:** Dihydrotestosterone; **EMT:** Epithelial–mesenchymal transition; **EPI-001:** EPI-001 androgen receptor N-terminal domain inhibitor; **ER:** Estrogen receptor; **HCC:** Hepatocellular carcinoma; **JAK-STAT:** Janus kinase–signal transducer and activator of transcription; **LBD:** Ligand-binding domain; **NF-κB:** Nuclear factor kappa B; **PARP:** Poly(ADP-ribose) polymerase; **PRPF6:** Pre-mRNA processing factor 6; **RAF/MEK/ERK:** Rapidly accelerated fibrosarcoma/Mitogen-activated protein kinase kinase/Extracellular signal-regulated kinase; **S-phase:** DNA synthesis phase of the cell cycle; **TNBC:** Triple-negative breast cancer.

**Supplementary Table 4: Summary of *AR-V7* and wild-type AR Expression in Hepatocellular Carcinoma Cell Lines**

| **No.** | **Cell line** | **Wild-Type AR Status** | ***AR-V7* Status** |
| --- | --- | --- | --- |
| 1 | HCCLM3 | + | + |
| 2 | HepG2 | - | - |
| 3 | HepG2/C3A | - | - |
| 4 | Huh7 | - | - |
| 5 | PLC5 | - | - |
| 6 | PLC/PRF/5 | - | - |
| 7 | SMMC-7721 | - | - |
| 8 | SNU-423 | + | + |
| 9 | SNU-475 | - | + |

**+:** positive; **–:** negative; **AR**: androgen receptor; ***AR-V7***: androgen receptor splice variant 7.

**Supplementary Table 5: Summary of *AR-V7* and wild-type AR Expression, and Hormone Receptor Status Across Breast Cancer Cell Lines**

| **No.** | **Cell line** | **Wild-Type AR Status** | ***AR-V7* Status** | **ER status** | **PR status** | **HER2 status** |
| --- | --- | --- | --- | --- | --- | --- |
| 1 | BT474 | + | + | + | + | OE |
| 2 | BT549 | - | - | - | - | - |
| 3 | CAL-51 | + | - | - | - | - |
| 4 | CAL-148 | + | + | - | - | - |
| 5 | HCC38 | - | - | - | - | - |
| 6 | HCC202 | + | + | - | - | + |
| 7 | HCC1954 | - | - | - | - | + |
| 8 | HCC1419 | + | + | +/- | - | + |
| 9 | Hs578T | - | - | - | - | - |
| 10 | LLC2 | + | + | LLC2 is a Tamoxifen-resistant MCF-7. | | |
| 11 | MCF7 | + | + | + | + | - |
| 12 | MDA-MB-453 | + | + | - | - | + |
| 13 | MDA-MB-231 | + | + | - | - | - |
| 14 | MDA-MB-361 | + | + | + | +/- | + |
| 15 | MDA-MB-468 | - | - | - | - | - |
| 16 | MDA-MB-157 | - | - | - | - | - |
| 17 | MFM223 | + | + | - | - | - |
| 18 | SK-BR-3 | - | - | - | - | + |
| 19 | T47D | + | + | + | + | - |
| 20 | UACC-812 | - | - | + | +/- | + |
| 21 | UACC-893 | + | + | - | - | + |
| 22 | ZR75.1 | + | + | + | +/- | - |

**+:** positive; **–:** negative; **AR**: androgen receptor; ***AR-V7***: androgen receptor splice variant 7; **ER**: estrogen receptor; **HER2**: human epidermal growth factor receptor 2; **OE**: overexpression; **PR**: progesterone receptor. **Note:** ER, PR, and HER2 receptor status for breast cancer cell lines were retrieved from previously published data (35)

**References:**

1.	Hu DG, Hickey TE, Irvine C, Wijayakumara DD, Lu L, Tilley WD, et al. Identification of androgen receptor splice variant transcripts in breast cancer cell lines and human tissues. Horm Cancer. 2014;5(2):61-71.

2.	Hickey TE, Irvine CM, Dvinge H, Tarulli GA, Hanson AR, Ryan NK, et al. Expression of androgen receptor splice variants in clinical breast cancers. Oncotarget. 2015;6(42):44728-44.

3.	Asano Y, Kashiwagi S, Goto W, Kurata K, Morisaki T, Noda S, et al. Abstract 3939: Clinical significance of expression of androgen-receptor splice variant-7 (AR-V7) in neoadjuvant chemotherapy for breast cancer. Cancer Research. 2016;76(14_Supplement):3939-.

4.	Singh B, Deng F-M, Kane Y, Zhan Y, Qi Y, Liu X, et al. Abstract P3-05-08: High prevalence of splicing variant AR-V7 in triple negative breast carcinoma. Cancer Research. 2016;76(4_Supplement):P3-05-8-P3--8.

5.	Stossi F, Dandekar RD, Bolt MJ, Newberg JY, Mancini MG, Kaushik AK, et al. High throughput microscopy identifies bisphenol AP, a bisphenol A analog, as a novel AR down-regulator. Oncotarget. 2016;7(13):16962-74.

6.	Aceto N, Bardia A, Wittner BS, Donaldson MC, O'Keefe R, Engstrom A, et al. AR Expression in Breast Cancer CTCs Associates with Bone Metastases. Mol Cancer Res. 2018;16(4):720-7.

7.	Braga DL, Mota STS, Zóia MAP, Lima P, Orsolin PC, Vecchi L, et al. Ethanolic Extracts from Azadirachta indica Leaves Modulate Transcriptional Levels of Hormone Receptor Variant in Breast Cancer Cell Lines. Int J Mol Sci. 2018;19(7).

8.	Kasimir-Bauer S, Keup C, Hoffmann O, Hauch S, Kimmig R, Bittner AK. Circulating Tumor Cells Expressing the Prostate Specific Membrane Antigen (PSMA) Indicate Worse Outcome in Primary, Non-Metastatic Triple-Negative Breast Cancer. Front Oncol. 2020;10:1658.

9.	Lehmann BD, Abramson VG, Sanders ME, Mayer EL, Haddad TC, Nanda R, et al. TBCRC 032 IB/II Multicenter Study: Molecular Insights to AR Antagonist and PI3K Inhibitor Efficacy in Patients with AR(+) Metastatic Triple-Negative Breast Cancer. Clin Cancer Res. 2020;26(9):2111-23.

10.	Ferguson DC, Mata DA, Tay TK, Traina TA, Gucalp A, Chandarlapaty S, et al. Androgen receptor splice variant-7 in breast cancer: clinical and pathologic correlations. Mod Pathol. 2022;35(3):396-402.

11.	Ali B, El-Abhar H, Abdallah D, Mohamed G, Sharaky M, Shouman S, et al. The Enzalutamide and EPI-001 modulate cell proliferation and metastasis markers in T47D by targeting AR/ARV7. Indonesian Journal of Pharmacy. 2023.

12.	Asemota S, Effah W, Young KL, Holt J, Cripe L, Ponnusamy S, et al. Identification of a targetable JAK-STAT enriched androgen receptor and androgen receptor splice variant positive triple-negative breast cancer subtype. Cell Rep. 2023;42(12):113461.

13.	Gatalica Z, Vranic S, Hall D, Hoag J, Thakkar S, Basu G. Androgen Receptor Splice Variant-7 is an Early Pathogenic Event in Breast Carcinomas with Apocrine Differentiation. Laboratory Investigation. 2024;104:S160-61.

14.	Kuroiwa Y, Ito K, Nakayama J, Semba K, Yamamoto Y. Analysis of the responsiveness to antiandrogens in multiple breast cancer cell lines. Genes Cells. 2024;29(4):301-15.

15.	Srivastava TP, Ajmeriya S, Goel I, Talukdar J, Srivastava A, Parshad R, et al. Prognostic role of Androgen Receptor splice variant 7 (AR-V7) in the pathogenesis of breast cancer. BMC Cancer. 2024;24(1):1398.

16.	Ali BM, El-Abhar HS, Mohamed G, Nassar HR, Aliedin N, Sharaky M, et al. A study of the role of androgen receptor and androgen receptor variant 7 in TNBC patients and the effect of their targeting by Enzalutamide and EPI-001 in MDA-MB-231. J Steroid Biochem Mol Biol. 2025;245:106636.

17.	Gatalica Z, Ye J, Chen Y-Y, Baehner R, Kimbrough J, Rose I, et al. Uncovering AR-v7 as a Common Early Event in Apocrine Breast Tumors: Rethinking Antiandrogen Therapy Strategies. Archives of Pathology & Laboratory Medicine. 2025;149:e238-9.

18.	Bian W, Zhang B, Song Z, Knisbacher BA, Chan YM, Bao C, et al. SplitFusion enables ultrasensitive gene fusion detection and reveals fusion variant-associated tumor heterogeneity. Patterns (N Y). 2025;6(2):101174.

19.	Mitani Y, Rao PH, Maity SN, Lee YC, Ferrarotto R, Post JC, et al. Alterations associated with androgen receptor gene activation in salivary duct carcinoma of both sexes: potential therapeutic ramifications. Clin Cancer Res. 2014;20(24):6570-81.

20.	Dalin MG, Desrichard A, Katabi N, Makarov V, Walsh LA, Lee KW, et al. Comprehensive Molecular Characterization of Salivary Duct Carcinoma Reveals Actionable Targets and Similarity to Apocrine Breast Cancer. Clin Cancer Res. 2016;22(18):4623-33.

21.	Li J, Mitani Y, Rao PH, Perlaky L, Liu B, Weber RS, et al. Establishment and genomic characterization of primary salivary duct carcinoma cell line. Oral Oncol. 2017;69:108-14.

22.	Cappelletti V, Miodini P, Reduzzi C, Alfieri S, Daidone MG, Licitra L, et al. Tailoring treatment of salivary duct carcinoma (SDC) by liquid biopsy: ARv7 expression in circulating tumor cells. Ann Oncol. 2018;29(7):1599-601.

23.	Kang H, Antonarakis ES, Luo J, Zheng Q, Rooper L, De Marzo AM, et al. Detection of AR-V7 transcript with RNA in situ hybridization in human salivary duct cancer. Oral Oncol. 2018;84:134-6.

24.	Gargano SM, Senarathne W, Feldman R, Florento E, Stafford P, Swensen J, et al. Novel therapeutic targets in salivary duct carcinoma uncovered by comprehensive molecular profiling. Cancer Med. 2019;8(17):7322-9.

25.	Yang RK, Zhao P, Lu C, Luo J, Hu R. Expression pattern of androgen receptor and AR-V7 in androgen-deprivation therapy-naïve salivary duct carcinomas. Hum Pathol. 2019;84:173-82.

26.	van Boxtel W, Verhaegh GW, van Engen-van Grunsven IA, van Strijp D, Kroeze LI, Ligtenberg MJ, et al. Prediction of clinical benefit from androgen deprivation therapy in salivary duct carcinoma patients. Int J Cancer. 2020;146(11):3196-206.

27.	Schvartsman G, Bell D, Rubin ML, Tetzlaff M, Hanna E, Lee JJ, et al. The tumor immune contexture of salivary duct carcinoma. Head Neck. 2021;43(4):1213-9.

28.	Sarıkaya EA, Korhan P, Incir C, Yıldız AH, Deger DM, Özer SM, et al. Evaluation of androgen receptor and androgen receptor splice-variant 7 in bladder cancer; a novel approach into an ancient topic. World J Urol. 2024;42(1):459.

29.	Dauki AM, Blachly JS, Kautto EA, Ezzat S, Abdel-Rahman MH, Coss CC. Transcriptionally Active Androgen Receptor Splice Variants Promote Hepatocellular Carcinoma Progression. Cancer Res. 2020;80(3):561-75.

30.	Song H, Sun N, Lin L, Wei S, Zeng K, Liu W, et al. Splicing factor PRPF6 upregulates oncogenic androgen receptor signaling pathway in hepatocellular carcinoma. Cancer Sci. 2020;111(10):3665-78.

31.	Gatalica Z, Vranic S, Krušlin B, Poorman K, Stafford P, Kacerovska D, et al. Comparison of the biomarkers for targeted therapies in primary extra-mammary and mammary Paget's disease. Cancer Med. 2020;9(4):1441-50.

32.	Kruslin B, Gatalica Z, Hes O, Skenderi F, Miettinen M, Contreras E, et al. TERT Gene Fusions Characterize a Subset of Metastatic Leydig Cell Tumors. Clin Genitourin Cancer. 2021;19(4):333-8.

33.	Wilson B, Xiu J, Ulm M, Redfern T, Narayanan R, ElNaggar A. Expression of androgen receptor splice variant, AR-V7, in high-grade serous ovarian cancer. Gynecologic Oncology. 2021;162:S157-S8.

34.	Wang L, Vasudevaraja V, Tran I, Sukhadia P, Reuter VE, Abu-Rustum NR, et al. Novel Androgen Receptor Splice Variant 7 in Gynecologic Tumors. Int J Gynecol Pathol. 2025;44(1):88-93.

35.	Dai X, Cheng H, Bai Z, Li J. Breast Cancer Cell Line Classification and Its Relevance with Breast Tumor Subtyping. J Cancer. 2017;8(16):3131-41.
