# Supplementary material for: The prevalence of the AR-V7 variant and its association with clinicopathologic characteristics in non-prostatic cancers—a systematic review
Source: Carcinogenesis. 2026 Aug 4;47(3):bgag052. doi: 10.1093/carcin/bgag052 (PMC13475121; doi:10.1093/carcin/bgag052)
Supplement: Supplementary file 1 [file bgag052_supplementary_data.zip › Supplementary file S2.docx]

**PubMed (October 30, 2025)**

**Search #1 search terms for *AR-V7* (n= 850)**

"AR-V7" OR "androgen receptor variant 7" OR "ARV7" OR "androgen receptor splice variant 7" OR "AR splice variant 7" OR "AR variant 7" OR "androgen receptor variant-7" OR "androgen receptor splice variant-7" OR "ARV-7"

**Search #2 search terms for non-prostatic cancers (n= 6,109,519)**

Cancer* OR carcinoma* OR tumor* OR neoplasm* OR malignan* OR melanoma* OR lymphoma* OR leukemia* OR glioma* OR cholangiocarcinoma* OR retinoblastoma* OR sarcoma* OR CML OR "multiple myeloma*" OR "neuroendocrine tumor*" OR fibrosarcoma* OR osteosarcoma* OR rhabdomyosarcoma* OR chondrosarcoma* OR leiomyosarcoma* OR liposarcoma* OR angiosarcoma* OR "gastrointestinal stromal" OR GIST OR myxofibrosarcoma* OR dermatofibrosarcoma* OR "malignant peripheral nerve"

**Search #3- Search #1 AND #2 (n= 843)**

**SCOPUS (October 30, 2025)**

**Search #1- search terms for *AR-V7* (n= 848)**

"AR-V7" OR "androgen receptor variant 7" OR "ARV7" OR "androgen receptor splice variant 7" OR "AR splice variant 7" OR "AR variant 7" OR "androgen receptor variant-7" OR "androgen receptor splice variant-7" OR "ARV-7"

## **Search #2 search terms for non-prostatic cancers (n= 7,357,048)**

Cancer* OR carcinoma* OR tumor* OR neoplasm* OR malignan* OR melanoma* OR lymphoma* OR leukemia* OR glioma* OR cholangiocarcinoma* OR retinoblastoma* OR sarcoma* OR CML OR "multiple myeloma*" OR "neuroendocrine tumor*" OR fibrosarcoma* OR osteosarcoma* OR rhabdomyosarcoma* OR chondrosarcoma* OR leiomyosarcoma* OR liposarcoma* OR angiosarcoma* OR "gastrointestinal stromal" OR GIST OR myxofibrosarcoma* OR dermatofibrosarcoma* OR "malignant peripheral nerve"

**Search #3- Search #1 AND #2 (n= 840)**

**Web of Science (October 30, 2025)**

**Search #1- search terms for *AR-V7* (n= 1058)**

"AR-V7" OR "androgen receptor variant 7" OR "ARV7" OR "androgen receptor splice variant 7" OR "AR splice variant 7" OR "AR variant 7" OR "androgen receptor variant-7" OR "androgen receptor splice variant-7" OR "ARV-7"

## **Search #2 search terms for non-prostatic cancers (n= 6,649,349)**

Cancer* OR carcinoma* OR tumor* OR neoplasm* OR malignan* OR melanoma* OR lymphoma* OR leukemia* OR glioma* OR cholangiocarcinoma* OR retinoblastoma* OR sarcoma* OR CML OR "multiple myeloma*" OR "neuroendocrine tumor*" OR fibrosarcoma* OR osteosarcoma* OR rhabdomyosarcoma* OR chondrosarcoma* OR leiomyosarcoma* OR liposarcoma* OR angiosarcoma* OR "gastrointestinal stromal" OR GIST OR myxofibrosarcoma* OR dermatofibrosarcoma* OR "malignant peripheral nerve"

**Search #3- Search #1 AND #2 (n= 1,029)**

**CINAHL ultimate (October 30, 2025)**

**Search #1- search terms for *AR-V7* (n= 112)**

"AR-V7" OR "androgen receptor variant 7" OR "ARV7" OR "androgen receptor splice variant 7" OR "AR splice variant 7" OR "AR variant 7" OR "androgen receptor variant-7" OR "androgen receptor splice variant-7" OR "ARV-7"

## **Search #2 search terms for non-prostatic cancers (n= 1,015,340)**

Cancer* OR carcinoma* OR tumor* OR neoplasm* OR malignan* OR melanoma* OR lymphoma* OR leukemia* OR glioma* OR cholangiocarcinoma* OR retinoblastoma* OR sarcoma* OR CML OR "multiple myeloma*" OR "neuroendocrine tumor*" OR fibrosarcoma* OR osteosarcoma* OR rhabdomyosarcoma* OR chondrosarcoma* OR leiomyosarcoma* OR liposarcoma* OR angiosarcoma* OR "gastrointestinal stromal" OR GIST OR myxofibrosarcoma* OR dermatofibrosarcoma* OR "malignant peripheral nerve"

**Search #3- Search #1 AND #2 (n= 107)**

**Cochrane Library (October 30, 2025)**

**Search #1- search terms for *AR-V7* (n= 68)**

"AR-V7" OR "androgen receptor variant 7" OR "ARV7" OR "androgen receptor splice variant 7" OR "AR splice variant 7" OR "AR variant 7" OR "androgen receptor variant-7" OR "androgen receptor splice variant-7" OR "ARV-7"

## **Search #2 search terms for non-prostatic cancers (n= 326233)**

Cancer* OR carcinoma* OR tumor* OR neoplasm* OR malignan* OR melanoma* OR lymphoma* OR leukemia* OR glioma* OR cholangiocarcinoma* OR retinoblastoma* OR sarcoma* OR CML OR "multiple myeloma*" OR "neuroendocrine tumor*" OR fibrosarcoma* OR osteosarcoma* OR rhabdomyosarcoma* OR chondrosarcoma* OR leiomyosarcoma* OR liposarcoma* OR angiosarcoma* OR "gastrointestinal stromal" OR GIST OR myxofibrosarcoma* OR dermatofibrosarcoma* OR "malignant peripheral nerve"

**Search #3- Search #1 AND #2 (n= 67)**

**European Society for Medical Oncology (ESMO)** **(October 30, 2025)**

Search terms for ***AR-V7*** manually (n=20)

**The American Society of Clinical Oncology (ASCO)** **(October 30, 2025)**

Search terms for ***AR-V7*** manually (n=86)

**American Association for Cancer Research (AACR) (October 30, 2025)**

**Search #1- search terms for *AR-V7* (n= 926)**

"AR-V7" OR "androgen receptor variant 7" OR "ARV7" OR "androgen receptor splice variant 7" OR "AR splice variant 7" OR "AR variant 7" OR "androgen receptor variant-7" OR "androgen receptor splice variant-7" OR "ARV-7"

## **Search #2 search terms for non-prostatic cancers (n= 587297)**

Cancer* OR carcinoma* OR tumor* OR neoplasm* OR malignan* OR melanoma* OR lymphoma* OR leukemia* OR glioma* OR cholangiocarcinoma* OR retinoblastoma* OR sarcoma* OR CML OR "multiple myeloma*" OR "neuroendocrine tumor*" OR fibrosarcoma* OR osteosarcoma* OR rhabdomyosarcoma* OR chondrosarcoma* OR leiomyosarcoma* OR liposarcoma* OR angiosarcoma* OR "gastrointestinal stromal" OR GIST OR myxofibrosarcoma* OR dermatofibrosarcoma* OR "malignant peripheral nerve"

**Search #3- Search #1 AND #2 (n= 27)**

**USCAP (October 30, 2025)**

Due to search functionality limitations of the USCAP meeting archive, which restricted the number of Boolean operators that could be used in a single query, only the *AR-V7* search terms (“AR-V7” OR “androgen receptor variant 7” OR “ARV7” OR “androgen receptor splice variant 7” OR “AR splice variant 7” OR “AR variant 7” OR “androgen receptor variant-7” OR “androgen receptor splice variant-7” OR “ARV-7”) were used. This search retrieved 15 results. All retrieved abstracts were manually screened, and 6 abstracts reporting *AR-V7* in non-prostatic cancers met the eligibility criteria and were extracted.
